# Six years review of the occupational exposure to blood and body fluids program at King Abdulaziz Medical City in the Western Region (KAMC-WR)

**DOI:** 10.1186/1753-6561-5-S6-P227

**Published:** 2011-06-29

**Authors:** N Nafouri, A Thaqafi, N Nafouri, M Mutasim, B Subyei, M Janadi

**Affiliations:** 1 Infection Prevention and Control, King Abdulaziz Medical City - WR, National Guard, Jeddah, Saudi Arabia

## Introduction / objectives

Healthcare providers are vital asset of KAMC-WR. Due to the nature of tasks they perform, the risk of exposure to sharps contaminated with blood and body fluids is high. The objective of this study is to review gathered data, assess the effectiveness of measures taken in reducing percutaneous injuries (PI) rate, and identify areas of potential improvement.

## Methods

PI rerecords from 2005 to 2010 were tracked using the Exposure Prevention Information Network (EPINet) program as adopted benchmark. Findings were analyzed using Root-Cause-Analysis (RCA) quality tool.

## Results

A total of 290 PI were reported during a cumulative total of 1797 average daily census. Steady reduction observed from 20.5/100 occupied beds in 2005 to 10.6 in 2009. The average rate in six years was 16.2. Even though PI rate increased to 18.1 in 2010 due to the safety culture survey conducted in 2009 to increase reporting by Medical Doctors (MD) still these rates were comparable to EPINET average rate of 2007 (27.97/100 occupied beds). The source was identified in 80% of reported PI. A total of 42 working days were lost. 72% of PI reported by females compared to 28% by males. Nurses and paramedical staff were the highest affected groups with exception in 2010 where MD ranked second. Incidents occurred during administration were found to be the highest contributing factor to PI followed by uncapped needles post administration and thirdly came improper discard of sharps.

## Conclusion

The program improved by 2 fold. EPINet program is a sounding approach to engage different occupation groups and MD in particular since decisions made were data driven. The need for comprehensive studies at bed-side, updating PI form, supporting closed systems and introducing focused training programs were identified as areas for improvement.

## Disclosure of interest

None declared.

